# Thrombotic Thrombocytopenic Purpura in Black People: Impact of Ethnicity on Survival and Genetic Risk Factors

**DOI:** 10.1371/journal.pone.0156679

**Published:** 2016-07-06

**Authors:** Suella Martino, Mathieu Jamme, Christophe Deligny, Marc Busson, Pascale Loiseau, Elie Azoulay, Lionel Galicier, Frédéric Pène, François Provôt, Antoine Dossier, Samir Saheb, Agnès Veyradier, Paul Coppo, French Reference Center for Thrombotic Microangiopathies

**Affiliations:** 1 Service d’Hématologie, Hôpital Saint-Antoine, AP-HP, Paris, France; 2 Centre de Référence des Microangiopathies Thrombotiques, Hôpital Saint-Antoine, AP-HP, et UPMC Univ Paris 06, Paris, France; 3 Service de Médecine interne, CHU de Fort-de France, Fort-de-France, Martinique, France; 4 AP-HP, Laboratoire Jean Dausset d’Immunologie et d’Histocompatibilité & INSERM, UMRS 1160, Hôpital Saint Louis, Sorbonne Paris Cité, Paris, France; 5 Université Paris Diderot, Paris, France; 6 Service de Réanimation Médicale, Hôpital Saint-Louis, AP-HP, Paris, France; 7 Service d’Immunologie clinique, Hôpital Saint-Louis, AP-HP, Paris, France; 8 Service de Réanimation Médicale, Hôpital Cochin, AP-HP, Paris, France; 9 Université Paris 5, Paris, France; 10 Service de Néphrologie, Hôpital Albert Calmette, Lille, France; 11 Service de Médecine Interne, Hôpital Bichat, AP-HP, Paris, France; 12 Service de Médecine interne 1, Hôpital La Pitié-Salpêtrière, AP-HP, Paris, France; 13 Service d’Hématologie Biologique, Hôpital Lariboisière, AP-HP, Paris, France; 14 Sorbonne Université, UPMC Univ Paris 06, Paris, France; 15 Inserm U1009, Institut Gustave Roussy, Villejuif, France; Mario Negri Institute for Pharmacological Research and Azienda Ospedaliera Ospedali Riuniti di Bergamo, ITALY

## Abstract

Black people are at increased risk of thrombotic thrombocytopenic purpura (TTP). Whether clinical presentation of TTP in Black patients has specific features is unknown. We assessed here differences in TTP presentation and outcome between Black and White patients. Clinical presentation was comparable between both ethnic groups. However, prognosis differed with a lower death rate in Black patients than in White patients (2.7% versus 11.6%, respectively, *P =* .*04*). Ethnicity, increasing age and neurologic involvement were retained as risk factors for death in a multivariable model (*P <* .*05* all). Sixty-day overall survival estimated by the Kaplan-Meier curves and compared with the Log-Rank test confirmed that Black patients had a better survival than White patients (*P =* .*03*). Salvage therapies were similarly performed between both groups, suggesting that disease severity was comparable. The comparison of HLA-DRB1*11, -DRB1*04 and -DQB1*03 allele frequencies between Black patients and healthy Black individuals revealed no significant difference. However, the protective allele against TTP, HLA-DRB1*04, was dramatically decreased in Black individuals in comparison with White individuals. Black people with TTP may have a better survival than White patients despite a comparable disease severity. A low natural frequency of HLA-DRB1*04 in Black ethnicity may account for the greater risk of TTP in this population.

## Introduction

Thrombotic thrombocytopenic purpura (TTP) is a severe form of thrombotic microangiopathy (TMA) characterized by a profound thrombocytopenia, erythrocyte fragmentation and organ failure of variable severity. TTP results from an excessive systemic platelet aggregation caused by a severe deficiency in ADAMTS13, the protease specifically involved in the cleavage of von Willebrand factor large multimers [1]. In adults, severe ADAMTS13 deficiency results mostly from polyclonal autoantibodies [2]. These pathophysiological findings account for the efficacy of plasma-based treatment in TTP, which allows supplying ADAMTS13 deficiency. Immunomodulatory drugs aimed at depleting anti-ADAMTS13 antibody-producing B-lymphocytes are associated to standard treatment in an increasing number of cases [3, 4]. Those therapeutic strategies allowed improving outstandingly TTP prognosis, with current survival rates of 80–85% [5]. Despite this significant progress, death still occurs in up to 20% of patients, usually within the first days of management. Age, cerebral involvement and a very high LDH level were associated with more death [6]. Moreover, cardiac troponin-I (cTnI) was also strongly associated with death as well as refractoriness [7, 8].

Black African and Caribbean people are overrepresented in TTP registries [9–12], suggesting the involvement of specific genetic risk factors. In White people, the human leukocyte antigen (HLA) class II DRB1*11 and DQB1*03 alleles were found to be genetic risk factors associated with TTP, whereas HLA DRB1*04 was found protective [13–16]. Interestingly, other diseases were also found overrepresented in Black people, such as arterial hypertension, systemic lupus erythematosus and monoclonal gammopathies. Of note, it was shown that these diseases may be more severe in Black people than in White people, and may therefore require a specific management. Moreover, the involved pathophysiological mechanisms may be distinct from those observed in White people [17–20]. In TTP, works reported possible ethnic differences in early outcome; indeed, Black patients with TTP were reported to experience more exacerbations [21], deserving further investigation. Here, we asked whether TTP presented with specific clinical presentation, prognosis and HLA-related features according to ethnicity. More particularly, we focused on ethnicity as a possible prognostic factor.

## Patients and Methods

### Study Design

To assess whether TTP had specific characteristics in Black patients, we compared clinical presentation, outcome and HLA class II DRB1*11, DRB1*04 and DQB1*03 genotyping of TTP in Black patients (including African and Caribbean patients) to those of White patients. Patients of both groups were included during the same period of time from October, 2000 (date at which our registry was set up) to the date of the study design (October, 2013) and studied until death or durable remission. They were included non-selectively from intensive care units and departments of Internal medicine, Hematology, and Nephrology in 26 French centers. This study was approved by our institutional review board in accordance with the Declaration of Helsinki (CPP Ile-de-France V, Hôpital Saint-Antoine, Paris), and the French Data Protection Authority (“Commission Nationale Informatique et Libertés”, CNIL, authorization n°911539, and “Comité consultatif sur le traitement de l’information en matière de recherche dans le domaine de la santé”, CCTIRS, authorization n°11.537, Paris, France). Written informed consent was obtained from patients studied for HLA typing. For patients not explored for HLA typing and explored routinely, a verbal consent was obtained and reported in the patient’s record, in accordance with the local ethical committee.

### Data collection

Each patient’s epidemiologic, clinical and routine biological data were collected by a research study nurse, as previously reported [22], using a standardized form, and transferred to a computerized database. Cerebral involvement included focal deficiency, seizure, headache, vigilance disturbances (inadequate response) or coma (absence of response). Renal involvement was assessed by serum creatinine level and the estimated glomerular filtration rate (eGFR) by the Cockcroft method. ADAMTS13 activity and the search of anti-ADAMTS13 antibodies were performed as previously described [23].

### Patients and Treatment

All adult patients with features of microangiopathy associated with a severe acquired ADAMTS13 deficiency (< 10% of normal activity) and no other cause for cytopenias (cancer, chemotherapy or drug, transplantation, HIV infection) were enrolled. To avoid the influence of inadequate plasma volumes in survival and to focus on the prognostic value of patient characteristics on diagnosis, we excluded patients who were not managed according to standard recommendations [4, 11, 24]. Complete response, durable remission, exacerbations and relapses were previously defined [4]. In case of disease exacerbation or relapse, daily TPE was resumed, in association with rituximab as a second-line treatment [4]. Patients without features of active infection received steroids (1 mg/kg/d for 3 weeks).

### HLA typing

All patients samples were referred to the National reference Center for the exploration of HLA system (Laboratoire d'Immunologie et d'Histocompatibilité, Hôpital Saint-Louis, Paris, France). Allele frequencies of African patients were compared to those of healthy people originating from Burkina-Faso, Nigeria, Cameroun, Senegal, Guinee-Bissau and Mali. All Caribbean patients originated from Martinique and Guadeloupe islands. Allele frequencies of Caribbean patients were compared to those of healthy people from Martinique.

Given the limited number of patients with available DNA samples in the present study, we specifically assessed allele frequencies of HLA-DRB1*11, DRB1*04 and DQB1*03. HLA DRB1*11, DRB1*04 and DQB1*03 medium resolution typings were performed as previously detailed [14].

### Statistical Analysis

Quantitative variables were summarized by median (range) and compared by the Wilcoxon rank-sum test; categorical data was summarized as count (%) and compared by the chi-squared test or Fisher's exact test. Overall survival between both ethnic groups was estimated by the Kaplan-Meier curves and compared with the Log-Rank test. Findings were considered statistically significant at P-values *< 0*.*05*. Risk factors of early mortality were investigated by logistic regression. A univariable analysis was first carried out, and variables associated with mortality at the *P <* .*20* level were included in a backward multivariable analysis. Only variables associated at the .*05* level were kept in the final model.

HLA DRB1*11, DRB1*04 and DQB1*03 allelic frequencies were compared by Fisher exact test. Odds ratio (OR) was calculated for each risk factor and given with its 95% Fisher’s exact confidence intervals. A Wilcoxon rank-sum test was used to compare continuous variables. Only variables associated at a level < .05 were kept in the final model. SPSS v15 software was used for analysis.

## Results

### 1. Study group

From the considered study period, the Registry included 3400 adult patients with a diagnosis of TMA and available data. Among them, 420 were considered as having a TTP with an acquired/autoimmune severe ADAMTS13 deficiency fulfilling all inclusion criteria of the study; all were followed during at least 30 days after treatment initiation. Two hundred and twenty-four patients were White and 74 others were Black. The 122 remaining patients were of a different ethnic origin or of undetermined ethnic origin and were not included in the present study. Twenty-eight patients from the 298 included here died during the 30-day period of observation, corresponding to a death rate of 9.4% (95% CI [8%-15%]). The other patients were discharged alive.

### 2. Univariate analysis

**Fig 1:**
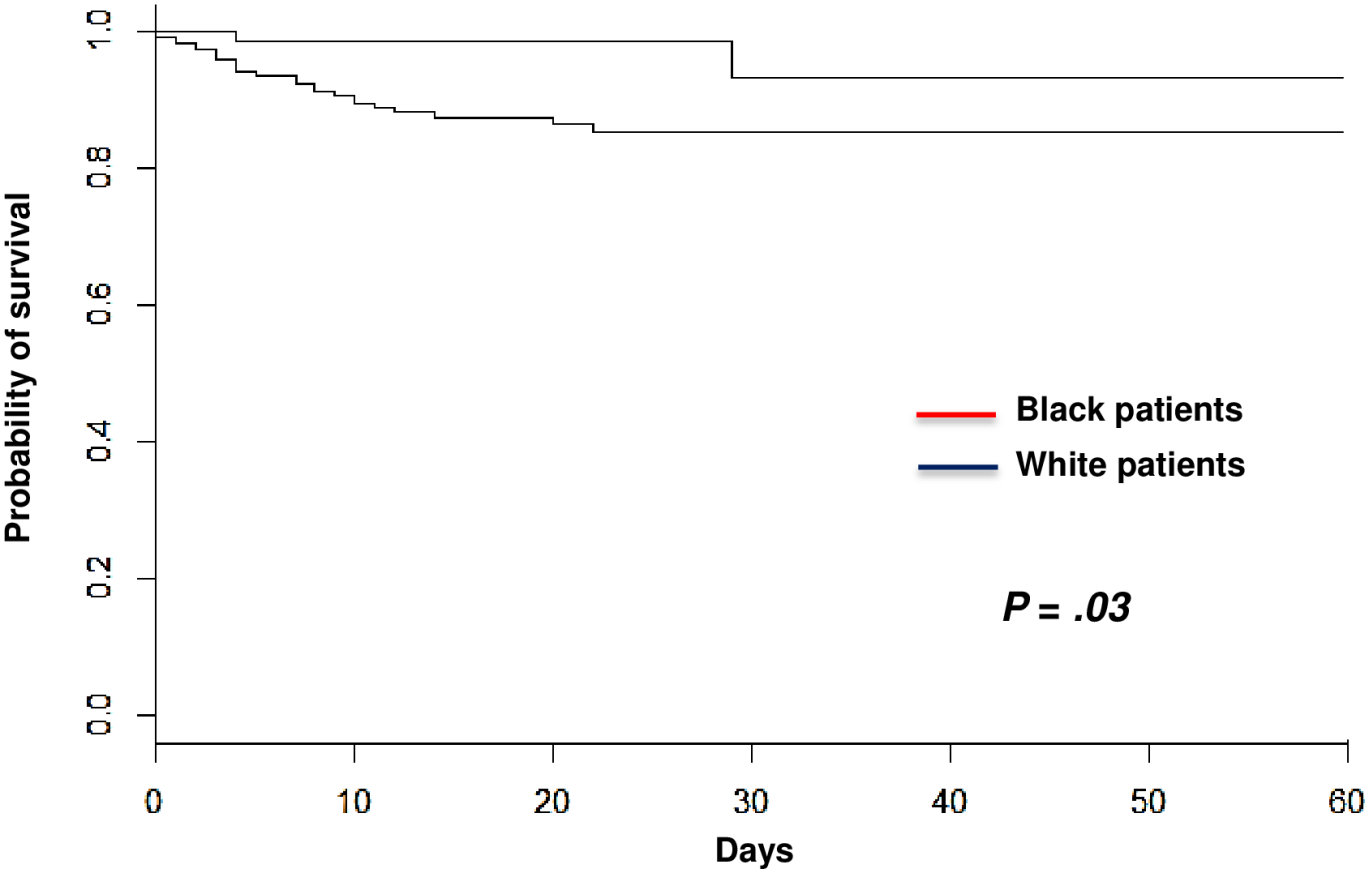
Kaplan-Meier survival estimates of overall survival in patients with TTP as a function of ethnicity (Black patients, n = 74; White patients, n = 224). Survival in both groups was compared with the Kaplan-Meier estimator.

Table 1 details clinical and biological characteristics of patients on diagnosis, treatment and outcome according to ethnicity. Clinical presentation and biological features on diagnosis were comparable between both groups. Particularly, age, cerebral involvement, LDH and cTnI levels were comparable, providing evidence that disease severity on diagnosis was comparable between Black and White patients. Steroids use, time to platelet count recovery and plasma volume required to achieve a durable complete remission were also comparable, and salvage therapies including rituximab (Table 1) were equally introduced in association with TPE. The number of flare-up episodes and relapse rate were also comparable. However, death rate was significantly lower in Black patients than in White patients. Indeed, only 2 Black patients died (2.7%) of multivisceral failure versus 26 in the White patients group (11.6%) (*P =* .*04*) (Table 1). This result was confirmed by calculation of 60-day overall survival estimated by the Kaplan-Meier curves and compared with the Log-Rank test (Fig 1). Black patients had a better survival than White patients (*P =* .*03*).

**Table 1:** Clinical characteristics of patients on diagnosis and outcome according to ethnicity.

	Black patients*	White patients	*P-value*
	(N = 74)	(N = 224)	
Age (years)	33 (26.2–49)	37 (27–49)	0.25
Sex (female)	57 (77)	149 (66.5)	0.12
Neurologic involvement	54 (73)	136 (60.7)	0.08
Hemoglobin level (g/dL)	7.35 (6.5–8.75)	7.7 (6.5–9.1)	0.95
Reticulocyte count (x10^9^/L)	169 (107–261)	169.6 (106–267)	0.30
Platelet count (x10^9^/L)	15.5 (8–23.7)	14 (9–21)	0.77
LDH level (IU/L)	5.75 (3.5–8.4)	4.89 (3–7)	0.15
Serum creatinine level (μmol/L)	101 (75–142)	91 (74.7–126)	0.18
Cardiac troponin Ic ≥ 0.25 μg/L	40%^1^	48%^2^	0.45
ANA (IU/mL)	33 (50.8)	115 (52.5)	0.92
Anti-dsDNA antibodies (IU/mL)	4 (12.5%)	20 (9.5)	0.53
Plasma volume (L/kg)	519 (277–1100)	599 (319–1044)	0.93
Steroids	68 (92%)	192 (86%)	0.23
Rituximab	31 (42%)	86 (38%)	0.58
Time to platelet count recovery (days)	16 (6–28.5)	18 (8–29)	0.26
Flare-up	39 (55)	113 (50.4)	0.6
Relapse	17 (23.6)	59 (26.3)	0.76
Mortality	2 (2.7)	26 (11.6)	**0.04**

Abbreviations: ANA: antinuclear antibodies. dsDNA: double-stranded deoxyribonucleic acid.

*Included Black Africans and Caribbeans.

^1^performed on 35

^2^54 patients.

Quantitative values are expressed in median (range) and compared by the Wilcoxon rank-sum test. Qualitative values are expressed as total number (%) and compared by the chi-squared test. *P<0*.*05* was considered statistically significant.

### 3. Multivariate analysis

Variables tested for inclusion in the model were ethnicity, age, neurologic involvement, LDH level, platelet count and serum creatinine level. Although cardiac cTnI assessment was performed randomly (as detailed in [8]), not all patients were explored, so this parameter could not be included in our model. Finally, ethnicity, increasing age and neurologic involvement were retained as risk factors in the multivariable model (OR = 0.2, 95% CI = 0.03–0.74; OR = 1.56, 95% CI = 1.19–2.6; OR = 3.43, 95% CI = 1.2–12.4, respectively, *P <* .*05* for all) (Table 2).

**Table 2:** Multivariate analysis.

	OR	*P-value*
Black ethnicity	0.2	**0.03**
Age	1.56	**0.002**
Neurologic involvement	3.43	**0.03**
Platelet count	0.99	0.67
LDH	1.06	0.09
Serum creatinine level (per 25 μmol/L)	1.03	0.62

OR: Odd-ratio. LDH: lactate dehydrogenase. *P<0*.*05* was considered statistically significant.

### 4. Human leukocyte antigen allele frequencies

The HLA alleles DRB1*11, DRB1*04 and DQB1*03 were studied in 26 African patients originating from 7 countries of West Africa, and 24 Caribbean patients. Accurate allele frequencies for HLA-DQB1*03 were not available for healthy individuals of Mali, Senegal and Guinea. Allele frequencies of HLA-DRB1*11, -DRB1*04 and -DQB1*03 between TTP patients and healthy individuals within Black ethnicity was comparable in all conditions (**Tables A to C** in S1 File). We also compared allele frequencies of HLA-DRB1*11, -DRB1*04 and -DQB1*03 alleles between Black individuals (patients and healthy people) and healthy White individuals. Interestingly, we observed that HLA-DRB1*11 allele frequency in Black people was comparable (Nigeria, Cameroon, Burkina-Faso) or increased (Mali, Senegal, Guinea) when compared to this observed in White people, whereas HLA-DQB1*03 allele frequency was comparable between both ethnic groups. More interestingly, the protective allele against autoimmune TTP, HLA-DRB1*04, was dramatically decreased in Black individuals (in TTP patients but also in healthy individuals) in comparison with White individuals (Table 3; **Tables D and E** in S1 File; http://www.allelefrequencies.net).

**Table 3:** Comparison of HLA-DRB1*04 allele frequencies between Black and White populations.

	HLA DRB1*04 allele			
	n	Frequency*	*P-value*	OR (95% CI)
Healthy individuals (White, N = 172)	48	0.16		
Autoimmune TTP (Africans, N = 26)	1	0.019	0.006	0.10 (0.0025–0.67)
Healthy individuals origin				
Nigeria (N = 258)	3	0.006	5.6x10^-18^	32.7 (0.006–0.098)
Cameroon (N = 126)	2	0.01	8x10^-11^	24 (6.01–206)
Burkina-Faso (N = 53)	0	0	5.2x10^-7^	-
Mali (N = 49)	4	0.04	0.004	4.33 (1.46–17.5)
Senegal (N = 112)	17	0.078	0.014	2.15 (1.13–4.27)
Guinea (N = 65)	10	0.077	0.06	2.12 (0.97–5)
Autoimmune TTP (Caribbeans, N = 24)	2	0.042	0.04	4.23 (0.98–38.5)
Healthy individuals origin				
Martinique (N = 100)	22	0.11	0.31	

HLA: human leukocyte antigen. OR: Odds ratio. CI: confidence interval. TTP: thrombotic thrombocytopenic purpura. N: total number of patients. n: number of patients bearing the allele.

*Allele frequency was calculated by dividing the number of patients bearing the allele with total number of alleles.

Comparison of phenotypic frequencies between both groups was performed using a 2-tailed Fisher exact test. *P<0*.*05* was considered statistically significant.

## Discussion

We report in this work that clinical presentation of autoimmune TTP was comparable between Black and White people. Response to treatment was also comparable for time to remission, exacerbation and relapse rate. However, we found that death rate was 4 times lower in Black people, with only two fatal outcomes in this group, including one patient with multiple risk factors of death (an old age, cerebral manifestations, an LDH level of 9.8 times the upper normal value and a cTnI level > 0.25 μg/L). Interestingly, despite this remarkable difference in death rate, disease severity was comparable between both groups; indeed, prognosis on diagnosis (including age, the prevalence of cerebral involvement, serum concentrations of LDH and cTnI) was comparable and salvage therapies were equally used in both ethnic groups. From these statements, one could hypothesize that Black people are more resistant to microthrombi-induced organ ischemia. Our results provide the first evidence that Black ethnicity, in addition to age and neurologic involvement is independently associated to survival.

This result was unexpected in this context since other vascular diseases such as arterial hypertension and malignant hypertension are of worse prognosis in Black people [25]. Moreover, Black people were reported to display physiologically higher levels of vWF than White people with, consequently, a lower ADAMTS13 activity [26]. However, one could hypothesize that in Black people this proagregant state is counterbalanced by other physiological mechanisms such as a more efficient fibrinolysis or a more efficient cleavage of vWF by alternative proteolytic pathways [27, 28], which may at least in part account for the better prognosis of TTP in Black people. In this work, we found that exacerbations were comparable between both ethnic groups, which is in contrast with a previous report that reported more exacerbation in African Americans [21]. However, this apparent discrepancy may be explained at least in part by different therapeutic modalities between both studies, which may have impacted on early outcome; indeed, TPE were usually interrupted progressively after remission achievement in one study, and immediately after remission achievement in the other. Moreover, a significant number of patients of one study received cyclosporine A.

Black people are more at risk of developing autoimmune TTP [9–11]. So far however, genetic risk factors involved in the occurrence of anti-ADAMTS13 antibodies in Black people are unknown. In White people, DRB1*11 and DQB1*03 alleles of the class II HLA system were identified as genetic risk factors of autoimmune TTP, whereas HLA DRB1*04 was found protective [13–16]. In this work, our results bring two conclusions. The first is that among Black individuals, there is no over-representation of DRB1*11 and DQB1*03 loci nor under-representation of DRB1*04 in patients with autoimmune TTP, suggesting additional risk factors for autoimmunity in Black people that remain to be discovered. The second, is the dramatically low natural frequency of the protective allele DRB1*04 in this ethnic group. This finding may therefore represent a strong risk factor for the development of autoimmune TTP in Black people, accounting for the over-representation of Black people in TTP registries. The reasons for which DRB1*04 is a protective allele for autoimmune TTP is still unknown. Recently, it was shown that DRB1*11 molecule recognizes CUB2 domain-derived peptides from ADAMTS13 with a higher efficiency than other HLA molecules [29], which may account for the increased occurrence of autoimmune TTP in patients harboring this HLA molecule. In the same manner, one could hypothesize that DRB1*04 could recognize ADAMTS13 epitopes with a poorer efficiency than the other HLA molecules, which may protect patients harboring this molecule from the disease.

Patients of our registry were managed homogeneously according to a written protocol based on previous studies and in accordance with international guidelines [4]. Therefore, we believe our results are robust. However, a major limitation of our study is the low number of events that were analyzed for some endpoints. This limitation is a general issue in TTP as it is a rare disease; it is also inherent in the questions raised by our work that led us to assess rare events (mortality and uncommon HLA alleles) in specific subgroups of patients. Consequently, our results need to be confirmed by other groups.

In conclusion, Black ethnicity may represent an independent prognostic factor associated with survival in TTP. Further studies from a larger number of patients are now needed to account for this observation. A low natural frequency of HLA-DRB1*04 in Black ethnicity may account for the greater risk of autoimmune TTP in this population, and studies are required to understand the protective mechanisms of this molecule.

## Supporting Information

S1 File **Tables A to C**. Comparison of HLA-DRB1*11 (**A**), -DRB1*04 (**B**) and -DQB1*03 (**C**) allele frequencies between TTP patients and healthy individuals within Black ethnicity. **Tables D and E**. Comparison of HLA-DRB1*11 (**D**) and HLA-DQB1*03 (**E**) allele frequencies between Black and White populations.
(DOCX)
