# Supplementary material for: Thrombotic Thrombocytopenic Purpura in Black People: Impact of Ethnicity on Survival and Genetic Risk Factors
Source: PLoS One. 2016 Jul 6;11(7):e0156679. doi: 10.1371/journal.pone.0156679 (PMC4934773; doi:10.1371/journal.pone.0156679)
Supplement: S1 File — Tables A to C. Comparison of HLA-DRB1*11 (A), -DRB1*04 (B) and -DQB1*03 (C) allele frequencies between TTP patients and healthy individuals within Black ethnicity. Tables D and E. Comparison of HLA-DRB1*11 (D) and HLA-DQB1*03 (E) allele frequencies between Black and White populations. (DOCX) [file pone.0156679.s001.docx]

**Supplemental Table A.** Comparison of HLA-DRB1*11 allele frequencies between Black patients and Black healthy individuals.

|  | HLA DRB1*11 allele | | |
| --- | --- | --- | --- |
|  | n | Frequency* | *P-value* |
| Autoimmune TTP (Africans, N=26) | 6 | 0.115 |  |
| Healthy individuals origin |  |  |  |
| Nigeria (N=258) | 77 | 0.15 | 0.65 |
| Cameroon (N=126) | 20 | 0.08 | 0.39 |
| Burkina-Faso (N=53) | 18 | 0.17 | 0.43 |
| Mali (N=49) | 21 | 0.21 | 0.13 |
| Senegal (N=112) | 40 | 0.177 | 0.26 |
| Guinea (N=65) | 25 | 0.193 | 0.22 |
|  |  |  |  |
| Autoimmune TTP (Caribbeans, N=24) | 6 | 0.125 |  |
| Healthy individuals origin |  |  |  |
| Martinique (N=100) | 32 | 0.16 | 0.66 |

HLA: human leukocyte antigen. TTP: thrombotic thrombocytopenic purpura. N: total number of patients. n: number of patients bearing the allele. *Allele frequency was calculated by dividing the number of patients bearing the allele with total number of alleles. Comparison of phenotypic frequencies between both groups was performed using a 2-tailed Fisher exact test.

**Supplemental Table B.** Comparison of HLA-DRB1*04 allele frequencies between Black patients and Black healthy individuals.

|  | HLA DRB1*04 allele | | |
| --- | --- | --- | --- |
|  | n | Frequency* | *P-value* |
| Autoimmune TTP (Africans, N=26) | 1 | 0.019 |  |
| Healthy individuals origin |  |  |  |
| Nigeria (N=258) | 3 | 0.006 | 0.32 |
| Cameroon (N=126) | 2 | 0.01 | 0.43 |
| Burkina-Faso (N=53) | 0 | 0 | 0.33 |
| Mali (N=49) | 4 | 0.08 | 0.65 |
| Senegal (N=112) | 17 | 0.078 | 0.19 |
| Guinea (N=65) | 10 | 0.077 | 0.17 |
|  |  |  |  |
| Autoimmune TTP (Caribbeans, N=24) | 2 | 0.042 |  |
| Healthy individuals origin |  |  |  |
| Martinique (N=100) | 22 | 0.11 | 0.18 |

HLA: human leukocyte antigen. TTP: thrombotic thrombocytopenic purpura. N: total number of patients. n: number of patients bearing the allele. *Allele frequency was calculated by dividing the number of patients bearing the allele with total number of alleles. Comparison of phenotypic frequencies between both groups was performed using a 2-tailed Fisher exact test or a chi-square test.

**Supplemental Table C.** Comparison of HLA-DQB1*03 allele frequencies between Black patients and Black healthy individuals.

|  | HLA DQB1*03 allele | | |
| --- | --- | --- | --- |
|  | n | Frequency* | *P-value* |
| Autoimmune TTP (Africans, N=26) | 17 | 0.32 |  |
| Healthy individuals origin |  |  |  |
| Nigeria (N=258) | - | - | - |
| Cameroon (N=126) | 58 | 0.232 | 0.09 |
| Burkina-Faso (N=53) | 24 | 0.23 | 0.10 |
|  |  |  |  |
| Autoimmune TTP (Caribbeans, N=24) | 12 | 0.25 |  |
| Healthy individuals origin |  |  |  |
| Martinique (N=100) | 50 | 0.25 | 1 |

HLA: human leukocyte antigen. TTP: thrombotic thrombocytopenic purpura. N: total number of patients. n: number of patients bearing the allele. *Allele frequency was calculated by dividing the number of patients bearing the allele with total number of alleles. Comparison of phenotypic frequencies between both groups was performed using a 2-tailed Fisher exact test.

**Supplemental Table D.** Comparison of HLA-DRB1*11 allele frequencies between Black and White populations.

|  | HLA DRB1*11 allele | | |  |
| --- | --- | --- | --- | --- |
|  | n | Frequency* | *P-value* | OR (95% CI) |
| Healthy individuals (White, N=172) | 39 | 0.12 |  |  |
| Autoimmune TTP (Africans, N=26) | 6 | 0.115 | 1 |  |
| Healthy individuals origin |  |  |  |  |
| Nigeria (N=258) | 77 | 0.15 | 0.12 |  |
| Cameroon (N=126) | 20 | 0.08 | 0.18 |  |
| Burkina-Faso (N=53) | 18 | 0.17 | 0.10 |  |
| Mali (N=49) | 21 | 0.21 | 0.006 | 2.54 (1.23-5.24) |
| Senegal (N=112) | 40 | 0.18 | 0.02 | 1.89 (1.08-3.31) |
| Guinea (N=65) | 25 | 0.19 | 0.02 | 2.12 (1.09-4.10) |
|  |  |  |  |  |
| Autoimmune TTP (Caribbeans, N=24) | 6 | 0.125 | 0.80 |  |
| Healthy individuals origin |  |  |  |  |
| Martinique (N=100) | 32 | 0.16 | 0.11 |  |

HLA: human leukocyte antigen. OR: Odds Ratio. CI: confidence interval. TTP: thrombotic thrombocytopenic purpura. N: total number of patients. n: number of patients bearing the allele. *Allele frequency was calculated by dividing the number of patients bearing the allele with total number of alleles. Comparison of phenotypic frequencies between both groups was performed using a 2-tailed Fisher exact test.

**Supplemental Table E.** Comparison of HLA-DQB1*03 allele frequencies between Black and White populations.

|  | HLA DQB1*03 allele | | |
| --- | --- | --- | --- |
|  | n | Frequency* | *P-value* |
| Healthy individuals (Caucasians, N=172) | 92 | 0.27 |  |
| Autoimmune TTP (Africans, N=26) | 17 | 0.32 | 0.29 |
| Healthy individuals origin |  |  |  |
| Cameroon (N=126) | 58 | 0.232 | 0.3 |
| Burkina-Faso (N=53) | 24 | 0.23 | 0.4 |
|  |  |  |  |
| Autoimmune TTP (Caribbeans, N=24) | 12 | 0.25 | 0.8 |
| Healthy individuals origin |  |  |  |
| Martinique (N=100) | 50 | 0.25 | 0.7 |

HLA: human leukocyte antigen. TTP: thrombotic thrombocytopenic purpura. N: total number of patients. n: number of patients bearing the allele. *Allele frequency was calculated by dividing the number of patients bearing the allele with total number of alleles. Comparison of phenotypic frequencies between both groups was performed using a 2-tailed Fisher exact test or a chi-square test.
